# Differences in Volatile Organic Compounds Between Concussed and Non-concussed Division I Athletes

**DOI:** 10.7759/cureus.61241

**Published:** 2024-05-28

**Authors:** Allyn Abadie, Ian McKeag, Dan Springer, Matthew H Hale, José R Fernández

**Affiliations:** 1 Department of Nutrition Sciences, University of Alabama Birmingham, Birmingham, USA; 2 Department of Family and Community Medicine, University of Alabama at Birmingham School of Medicine, Birmingham, USA; 3 Department of Athletics, University of Alabama Birmingham, Birmingham, USA

**Keywords:** breath, concussion recovery, ketone, sports related concussion, breath acetone, tramatic brain injury

## Abstract

Introduction

Diagnosing a concussion is challenging because of complex and variable symptoms. Establishing a viable biomarker of injury may rely on physiologic measurements rather than symptomology. Volatile organic compounds (VOCs) such as breath acetone have been identified as potential physiological markers that can capture changes in the utilization of energy substrates post-concussion. Here, we aimed to explore whether differences in VOCs exist between concussed and non-concussed athletes at the initial and later stages of injury recovery.

Methods

Six (N=6) non-concussed athletes were enrolled as control participants prior to the competitive season. Control participants’ breath acetone, heart rate, and anthropometric measures were obtained at rest and throughout a single exercise challenge by breathalyzer. Six (N=6) athletes diagnosed with concussion during the competitive season had breath acetone measured daily until cleared to return to activity or approximately four weeks following enrollment where they participated in an exit exercise challenge having breath acetone, heart rate, and anthropometric measures obtained. Comparisons were made between at-rest measures of concussed and non-concussed participants at multiple time points during the recovery period. Paired t-test comparisons with individuals serving as their own control were used to determine individual differences in recovery. Visual graphs were used to demonstrate differences in obtained measures amongst individuals and between groups during the exercise challenges.

Results

Results demonstrated statistically significant differences in breath acetone between concussed and control participants when the highest day measured during the first week of concussion was compared to the control participant’s resting values (P=0.017). Additionally, when the concussed participants served as their own control and their highest measured day of the first week post-concussion was compared to values when cleared to return to activity or at 26 days post-concussion, there was a significant difference in breath acetone (P=0.028). Comparing breath acetone during exercise between non-concussed and cleared concussed participants or four weeks post-injury, demonstrated no significant differences throughout the challenge or at rest prior. Visual graph comparisons in a single participant before and after concussion suggest differences may appear following exercise during the recovery period.

Discussion

These results suggest VOCs, particularly breath acetone, have the potential to serve as diagnostic markers of concussion. However, longitudinal research within larger cohorts and with equipment able to expel VOCs other than acetone from measures are needed to make informed recommendations.

## Introduction

Brain injuries caused by biomechanical forces, known as concussions, are complex due to their varied clinical presentation, including a wide range of signs and symptoms [1]. In National Collegiate Athletic Association (NCAA) sports, approximately 4.13 concussions occur for every 10,000 athlete-exposures [2]. The term concussion is widely known, as media coverage of the injury has increased in recent years. Yet, despite research efforts, a standardized objective diagnostic measurement has not been adopted. Thus, diagnosis of a concussion remains largely dependent on the clinical judgement of a medical provider with limited quantitative tools none of which is 100% sensitive or specific to inform decision-making [3]. A concussion is the most common type of mild traumatic brain injury and although labeled as mild, can lead to debilitating consequences such as permanent cognitive and physical disablement or death [4]. It is critical to establish a method to accurately diagnose concussions and restrict individuals from participating in potentially risky activities until fully recovered.

Current methods of diagnosis and return-to-play decision-making rely on clinician judgement and subjective testing methods. Many studies addressing concussion diagnosis use cognitive sideline assessments such as the Sport Concussion Assessment Tool (SCAT-5 which was recently updated to the SCAT-6), which is the most rigorous and well-established of the sideline assessments [1,5]. Standard return-to-play protocols gradually return an individual to activity as their symptoms resolve according to the SCAT-5 tool and its graded symptom checklist. The protocols involve introduction of exercise and symptom-provoking activities while under a designated symptom limit. The recent update to the assessment tool also ushered in a new era of injury management including six stages where continued progression through the early stages is possible if symptom increase is minimal [1]. To progress past the first three stages patients must have returned to their pre-concussed status. If they experience provocation of symptoms at any time in stages four through six, they must return to stage three. If an activity provokes a symptom, athletes are moved back one stage of the protocol [1]. Yet, this assessment has also been criticized by clinicians for the possible underreporting of symptoms due to the subjective nature of the tool [6]. Attempts have been made to strengthen diagnosis of concussion via the addition of objective markers of the physiologic consequences post-concussion rather than relying solely on signs and symptoms. For example, salivary small non-coding RNA markers with unique signatures have been shown to predict traumatic brain injury with 91% accuracy in males [7]. Additionally, using an algorithm that included age and 16 non-coding RNAs, researchers were able to predict persistent post-concussion symptoms with greater accuracy than by current methods (balance and cognitive testing) and demonstrated an added statistical benefit when the algorithm was combined with the current methods of symptom tracking [8]. Together, these prior studies illustrate the potential for physiologic markers to improve or perhaps even surpass current subjective assessment methods. However, tests for these markers are still not employable on a sideline.

The human breath contains hundreds of volatile organic compounds [9]; one of the most recognizable is ethanol which is often used by breathalyzers to translate blood alcohol content for criminal or civil matters [10]. Capturing changes in these gaseous biomarkers can help provide clinical diagnoses and enhance long-term monitoring of disease. Though most research connecting volatile organic compounds (VOCs) with pathologies investigate the diagnostic capability in respiratory disease, recent studies utilizing VOCs to differentiate other diseases such as cardiac, gastrointestinal, and neurological conditions as well as various cancers have appeared [9]. In a recent study published in Frontiers in Neurology, investigators used metabolomic profiling to compare military personnel subjected to repeat sub-concussive blasts (which produce concussive-like brain impairments) with those not subjected to the blasts and identified a combination of VOCs (acetic acid, acetone, and methanol) that accurately classified those subjected to the blasts with 98% accuracy [11]. However, these results have not yet been explored using clinical devices, such as breath acetone breathalyzers; which may be more useful and convenient for monitoring. Devices that are designed to measure a specific VOC, such as acetone or ethanol, are very sensitive but have difficulty differentiating between the intended VOC and the hundreds of others delivered in human breath with robust accuracy [9,10]. However, consistent use of a single device in clinical practice could offer information on the role and differentiating capability of VOCs during and after injury. The monitoring of VOCs offers impactful data to the scientific and clinical community, even without being able to differentiate singular compound influence.

Following concussion, a neurometabolic cascade of events is known to occur in humans and animal models [12]. The hallmark of this cascade is two stages of metabolic change. First, immediately following injury, neurotransmitters are released, and a hypermetabolic state occurs, resulting in increased use of glucose for energy and an accumulation of calcium in cerebral mitochondria [13]. In experimental animal models, this stage has been observed immediately and lasts up to 30 minutes after fluid percussion injury and up to four hours after cortical contusion [14]. Second, a more prolonged stage of glucose hypometabolism occurs as glycolysis is inhibited and the previous accumulation of calcium causes metabolic dysfunction in mitochondria [13]. This stage typically begins six hours following injury in humans, and its length is dependent on injury severity and age, but normally lasts approximately 14-28 days post injury in humans and five to 10 days in animal models [14,15]. Acetone, a ketone, is a byproduct of the utilization of other ketones for energy (acetoacetate and β-hydroxybutyrate) and can be measured portably and non-invasively through the exhaled breath due to its small size [16]. Acetone is considered a volatile organic compound, whereas the other ketone bodies are not. Acetone production and the overall use of ketones are regulated by glucose levels where low levels of glucose or dysfunction in the use of glucose triggers higher acetone production as an alternative energy substrate [17]. Thus, when glucose utilization is disrupted following a concussive head injury, the resultant increase in acetone production may serve as a detectable diagnostic marker of concussion when it is compared to concentrations in non-concussed individuals.

The overall objective of this project is to explore the potential of volatile organic compounds to serve as an objective diagnostic marker of concussion through measurements from a breath acetone breathalyzer device. The primary hypothesis of this study is that there will be a difference in device-measured breath acetone between concussed and non-concussed participants during injury presentation. In addition, full recovery following concussion implies that metabolism of energy substrates has normalized even when provoked during an exercise challenge, and thus the secondary hypothesis of this investigation is that there will be no differences in breath acetone during or following an exercise challenge between non-concussed individuals and concussed individuals cleared to return to activities or measured four weeks following concussion. Should there indeed be differences, it would suggest that the individuals had not returned to a normal metabolic state.

## Materials and methods

Experimental overview

**Figure 1:**
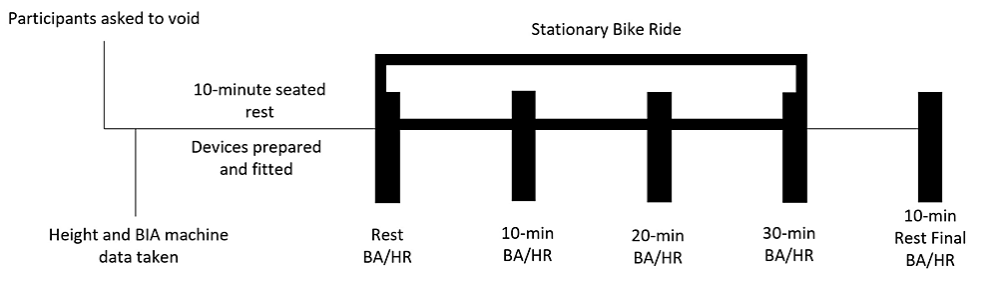
Exercise Challenge Protocol for Non-concussed and Post-concussion Participants Abbreviations: BIA (body impedance analysis), BA (breath acetone), HR (heart rate), min (minute)

This experiment is part of the larger Breath Acetone Concussion Ketone Study (BrACKS). Breath acetone, and subsequently other device-detected volatile organic compounds, were evaluated at a Division I university for their potential as biomarkers of concussion recovery through small sample exploratory analyses. Recent literature in metabolomics suggested VOCs could have a diagnostic role in concussion, yet they have not been evaluated in a concussed population using a portable clinical device. Prior to the competitive season, athletes from football and men and women’s soccer programs were recruited for baseline testing (no concussion present). Baseline testing, as seen in Figure 1, consisted of anthropometric measurements and an exercise challenge with multiple testing points in which breath acetone was measured with a portable ketone breathalyzer and heart rate measured with a chest sensor. The exercise challenge was a 30-minute stationary bike ride. Participants were tested at rest prior to the challenge, at 10 minutes of riding, at 20 minutes of riding, immediately after the challenge at 30 minutes of riding, and after 10 minutes of rest following the challenge.

During the competitive season, football and soccer athletes from the same Division I university who were diagnosed with a concussion and met inclusion/exclusion criteria were enrolled in the study and tested daily for breath acetone and thus VOC concentration. This testing was done in conjunction with the university standard post-concussion monitoring of athletes, which consisted of daily symptom monitoring with the SCAT-5 (symptom severity and symptom number). Daily resting breath acetone was measured while participants were seated following completion of the symptom checklist. Three breaths were taken consecutively, and an average calculated as the daily measurement. Given that it is not clear when metabolic changes in acetone or other VOCs occur and are detectable following a traumatic brain injury, multiple post-concussion measures were calculated and compared to a non-concussed sample, including at the first opportunity following concussion, the mean across the first week after concussion, and the highest day within the first week post-concussion.

Once a concussed athlete was cleared to participate in contact sport activity, the individual completed an exit test consisting of an exercise challenge also completed by non-concussed participants. If an athlete was not cleared to participate in contact sport activity within 28 days after daily measures had begun, they would also complete an exit test if sports medicine staff deemed the participant able to withstand the aerobic exercise protocol.

Participant exclusion criteria

Athletes were excluded from participation in the study if they were under 18 or older than 26 years old. Participants were excluded from baseline testing and/or post-injury testing if they were currently concussed, if they were following a low-carbohydrate or ketogenic diet, or if they had an underlying medical condition known to impact testing protocol or breath acetone concentration (e.g., diabetes, asthma, chronic neurological condition). To participate post-injury, participants had to be formally diagnosed with a concussion by the team physician and/or athletic trainer prior to enrollment.

Measurements

Breath Acetone

Breath acetone was measured in parts per million (ppm) by a portable breath analyzer able to detect measures 0-200 ppm (Ketonix Professional, Ketonix, Stockholm, Sweden). Acetone devices are limited, however, and though they give values of breath acetone, those values may be compounded by other VOCs such as methane or alcohol [9,10]. When describing measurements in terms of “breath acetone” there is acknowledgement by these researchers to the fact that other VOCs may be influencing the values. Therefore, readers should understand that any changes described as “breath acetone” will henceforth refer to VOCs as the authors cannot differentiate breath acetone concentration from the other gases based on the current state of portable devices.

The Ketonix device was chosen as the device for this study due to its standing as a registered Class I FDA medical device for the purposes of diet management and diabetes diagnosis [16,18]. As of January 2021, it was one of five devices that was FDA approved for such purposes [16]. Additionally, it has been used in multiple peer-reviewed human studies for the purpose of assessing breath acetone to influence adherence to a diabetes program [19], to detect ketosis in adults and children [20], and to relate to inter-day variation in appetite [21]. In the study on appetite variation, breath acetone measured by the Ketonix® device was also shown to correlate with fasting plasma lipopolysaccharide concentration and fasting plasma triglycerides [21].

In this study, breath acetone concentration was measured during the non-concussed baseline test and the concussed exit tests at five time points before, during, and after the exercise challenge (rest, at 10 minutes of exercise, at 20 minutes of exercise, at 30 minutes of exercise, and after 10 minutes of rest), as well as daily post-concussion. The first breath acetone measurement was taken after a 10-minute seated rest, and breath measurements were always completed with the participant in a seated position either in a chair or on a stationary bicycle. Breath acetone was also measured daily after an individual was diagnosed with a concussion and enrolled in the study. Measurements were taken while participants were in a seated position following a period of rest during which they filled out the symptom checklist portion of the SCAT-5. The method of breath collection followed device recommendations and a tidal breath technique (approx. 8 seconds) was utilized due to its ability to capture acetone concentration and its be conducted repeatedly over a short period [16,22].

Heart Rate

Heart rate was measured with a Polar FT4 chest sensor (Polar Electro, NY, USA) during the baseline and exit tests at the five time points. The device can detect heart rate values from 15 to 240 bpm and has an accuracy of ± 1% or ± 1 bpm (whichever is larger). The device was fitted to participants prior to the exercise challenges to ensure proper measurements were obtained. The first resting heart rate was taken after a 10-minute seated rest.

Anthropometrics

Height was measured by a stadiometer in centimeter units. An Omron HBF-514C bioimpedance scale (Omron, Kyoto, Japan) was used to measure body weight (lbs.), body mass index (BMI), body fat percentage (%), visceral fat level (relative level), and skeletal muscle percentage (%). Measurements were taken after the participant voided and before the initial rest leading to the exercise challenge in both baseline and exit tests.

Statistical Analysis

Statistical analyses were completed with XLSTAT v. 3.1 (Data Analysis and Statistical Solution for Microsoft Excel; Addinsoft, Paris, France, 2022). Scatter plot visuals were used to display and compare participants’ measurements during the exercise tests. The non-parametric Mann-Whitney U test was used to compare differences in concussed and non-concussed participants due to lack of normality at various time points (two days post-concussion, three days post-concussion, average of first week, and the day the highest value was measured during first week). Except for the comparison between non-concussed and the average of the first week as a concussed individual, each of the other time comparisons were 1:1 day comparisons. A Kruskal-Wallis test was completed to express differences between the previous comparisons (initial presentation at two days post-concussion and three days post-concussion, final presentation at clearance or four weeks post-concussion, the day with the highest value measured in the first week post-concussion, and non-concussed resting) and displayed with a Demsar plot. To address the hypothesis and account for individual variability, a parametric statistical approach was additionally used comparing the day with the highest value measured in the first week post-concussion to their own resting values taken when cleared to return to activity at the exit challenge or at 26 days post-concussion using a paired t-test. The value of 26 days post-concussion was selected due to its proximity to 28 days post-concussion and there were no missing values in the non-cleared participants that day. Statistical tests were limited due to the small sample sizes. Though statistical significance (P<0.05) was sought with these analyses, results suggesting close significance or strictly visual trends were also noted and discussed as they could inform future investigations with larger cohorts.

## Results

Participants

**Figure 2:**
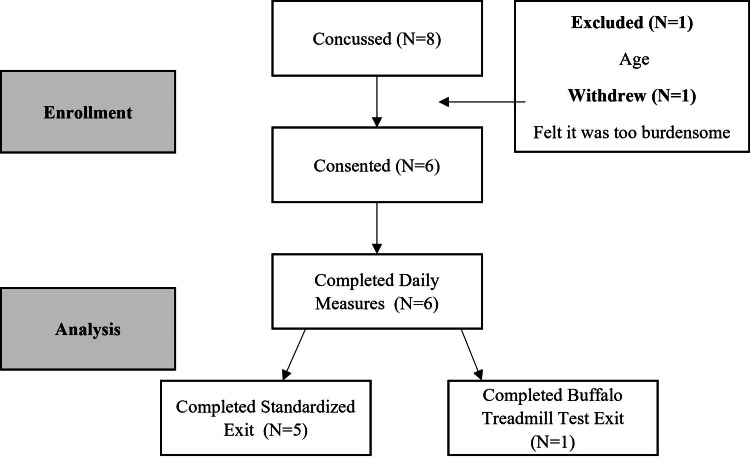
Breath Acetone Concussion Ketone Study (BrACKS) Consort Diagram

Characteristics of the non-concussed and concussed groups are presented in Table 1. Three female and three male soccer players who met the criteria for inclusion as control (non-concussed) participants were enrolled for baseline testing. Football athletes were not permitted to do baseline testing because at the time they were undergoing burdensome coronavirus disease 2019 (COVID-19) testing protocols and rigorous team and individual workout activities necessary to return to play after the pandemic-related disruption. During the competitive fall season, eight athletes were diagnosed with a concussion. Of these, one athlete did not meet inclusion criteria of age, and another withdrew from the study due to the perceived burden of required testing (Figure 2). The remaining six athletes with concussion completed daily testing for this study. One participant who completed a baseline test as a control participant was also among the six who sustained a concussion during the study investigation period.

**Table 1:** Participant Demographics

Category	Non-concussed	Concussed
Gender (N)		
Male	3	6
Female	3	0
Sport (N)		
Football	0	5
Men’s Soccer	3	1
Women’s Soccer	3	0
Age (yrs)		
Mean ± SD	20.3 ± 0.8	20.0 ± 1.1
Height (in)		
Mean ± SD	67.9 ± 4.4	74.0 ± 2.4
Weight (lbs)		
Mean ± SD	142.7 ± 23.2	241.3 ± 52.4
BMI (kg/m2)		
Mean ± SD	21.6 ± 1.08	30.6 ± 6.30
Race (N)		
Black	1	2
White	5	4

Control breath acetone

Breath acetone for control participants (N=6) was measured following a 10-minute seated rest prior to engaging in the exercise challenge. These values served as the primary comparison for the concussed athlete data. Within the control participants, resting breath acetone was measured at a range of 1.0 to 15.0 ppm for female participants (9.7 ± 7.7; mean ± SD) and at a range of 5.0 to 16.0 ppm for male participants (9.7 ± 5.7 ppm; mean ± SD) (male vs. female; P=1.00). Given that there was no statistical difference in non-concussed participants based on gender, the data from female athletes was retained for analyses.

Initial presentation breath acetone

**Figure 3:**
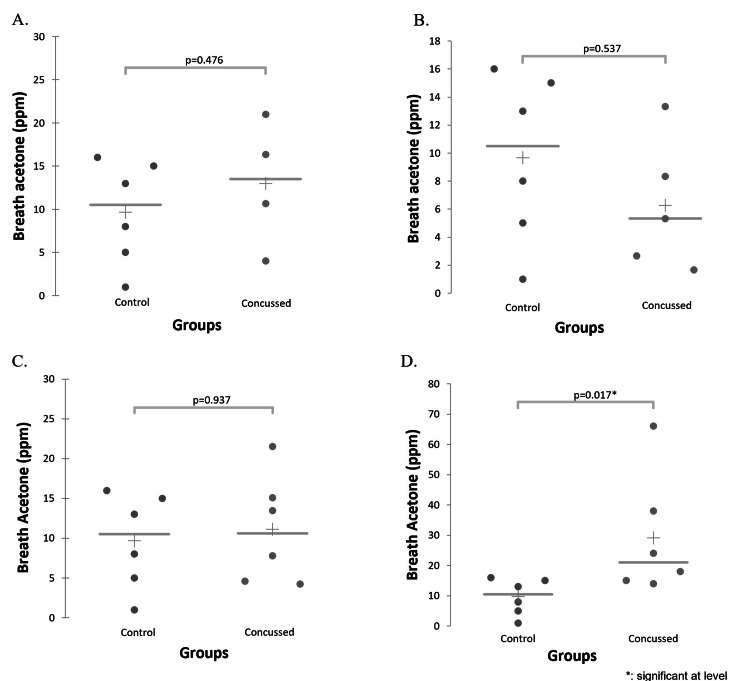
Differences in Breath Acetone Between Control and Concussed Participants Differences in breath acetone between control participants at rest and concussed participants two days post-injury (A), between control participants at rest and concussed participants three days post-injury (B), between control participants at rest and concussed participants’ average first seven days post-concussion (C), and control participants at rest and concussed participants’ highest recorded value in the first seven days post-concussion (D). Only the differences present in (D) demonstrated a statistically significant difference (P<0.05). Abbreviations: parts per million (ppm)

For post-concussion participants across their recovery, breath acetone was measured at a range of 0-186 ppm for the daily average of three subsequent breath tests (5.0 (8.9) ppm; median (Interquartile Range (IQR)). No players had breath acetone measured on the day of the concussion injury. Two concussed participants recorded initial breath acetone measurements on the first day following concussion, four recorded measurements on the second day, and five recorded measurements on the third day. To evaluate early-stage concussion presentation, data from the first day post-concussion were removed due to only two athletes having data that day. Mann-Whitney U comparisons of post-concussion participants with control values demonstrated no significant difference at two days post-injury (P=0.476), three days post-injury (P=0.537), or when comparing the average of the first seven days post-concussion (P=0.937; Figure 3A-3C). When using a Mann-Whitney U test to compare control participants with concussed participants’ highest measured value in the first seven days post-injury, there was a significant difference between concussed and control participants (P=0.017; Figure 3D).

**Figure 4:**
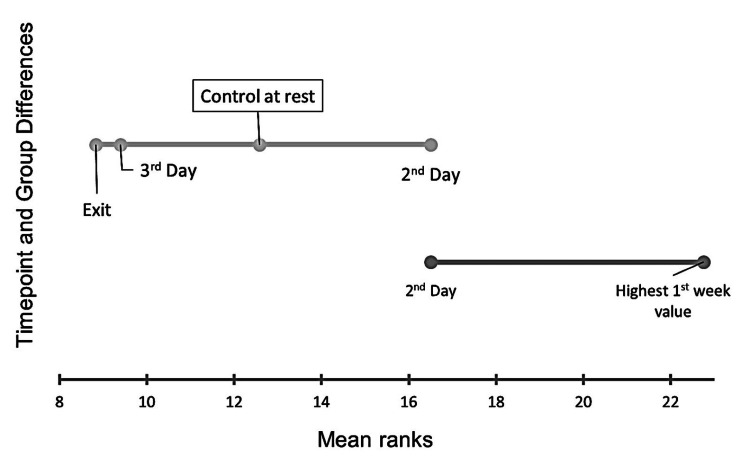
Demsar Plot of Differences in Breath Acetone Demsar mean ranks plot using the Conover-Iman multiple comparison procedure to show differences in breath acetone between concussed participants at various time points (exit test, third day post-concussion, second day post-concussion, and highest recorded value of the first week post-concussion) and control participants at rest.

Consistent with the results described above, results were the same when analyses were repeated using a Kruskal-Wallis test. The Kruskal-Wallis test was used to evaluate relationships among all measured and compared time points, specifically, all variables reflecting early-stage breath acetone (post-concussion day two, post-concussion day three, highest value recorded within the first seven days post-concussion, average of first week post-concussion) and resting value at clearance/exit test. These time points were compared in an analysis including the control resting values. Analysis demonstrated there were differences (P=0.017) between all the time points driven by the difference between the highest breath acetone concentration measured during the first week post-concussion and the non-concussed control group. The Demsar plot of the Kruskal-Wallis post-hoc multiple comparisons analysis visually displays the differences among the measured groups (Figure 4). Interestingly, significant differences between the highest measured value within the first week were found between that measure and the control participants (P=0.011), the concussed participants when cleared or four weeks post-concussion (P=0.001), and the third day following concussion (P=0.002) (Table 2). There was no statistically significant difference found when comparing the second day following concussion with the highest value measured in the first week (P=0.139).

**Table 2:** Post-Hoc Multiple Comparison: Breath Acetone at Multiple Time Points Significance is noted with * signifying P<0.05.

	Non-Concussed	Concussed Exit	Concussed 2nd Day Post	Concussed 3rd Day Post	Concussed Highest 1st Week
Non-Concussed	1	0.314	0.346	0.413	0.011*
Concussed Exit	0.314	1	0.073	0.883	0.001*
Concussed 2nd Day Post	0.346	0.073	1	0.107	0.139
Concussed 3rd Day Post	0.413	0.883	0.107	1	0.002*
Concussed Highest 1st Week	0.011*	0.001*	0.139	0.002*	1

Individual breath acetone

In the Demsar plot demonstrating the Kruskal-Wallis test comparisons (Figure 4), the resting breath acetone from non-concussed control individuals did not demonstrate a statistically significant difference when compared to the values measured at rest when the concussed individuals underwent the exit challenge (at clearance or approximately 28 days post-concussion). To control individual variation between concussed and non-concussed groups, a follow-up analysis was completed comparing the concussed individuals to themselves rather than an external group. To assess the ability of breath acetone to differentiate diagnostically, the resting value measured at the exit challenge (for participants cleared to return to activity) or at 26 days post-concussion (for non-cleared individuals) served as their control or "non-concussed" value. The control values of individuals were compared to their highest value measured in the first week post-concussion by a paired t-test similar to a pre-post analysis. There was found to be a statistically significant difference (P=0.028) indicating during the first week post-concussion breath acetone values can be differentiated from those measured when cleared to return to activity or 26-days post-concussion.

Exit test

The exit test was conducted when participants were cleared to return to contact athletic activities by the team physician, or at four weeks post-injury if medical staff thought the individual could complete the study protocol safely. One participant was unable to complete the exit exercise challenge due to severity of symptoms and instead completed a mild buffalo treadmill test (Figure 2). Although that participant was unable to be included in statistical tests involving the entirety of the exit exercise challenge, resting measures from that participant were used in comparisons involving post-concussion resting breath acetone.

**Figure 5:**
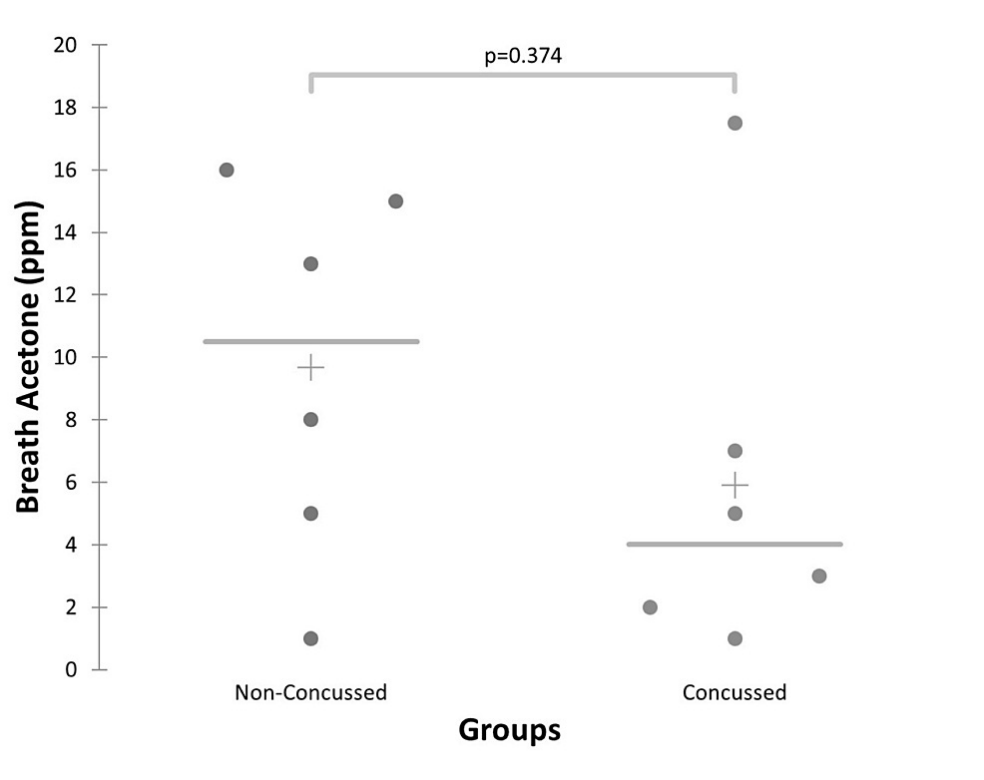
Resting Breath Acetone at Exercise Challenge Abbreviation: parts per million (ppm)

The resting values of the non-concussed participants were used as a control value for comparison with post-concussion participants who were tested throughout their concussion recovery and at the exit test. The control participants had resting breath acetone measured at a range of 1.0 to 16.0 (10.5 (8.8); median (IQR)). At rest prior to the exit exercise test, post-concussion participants’ breath acetone was measured at a range of 1.0 to 17.5 (3.0(5.0); median (IQR)). When these measures were compared to control participants, there was no statistically significant difference in breath acetone (P=0.374; Figure 5).

**Figure 6:**
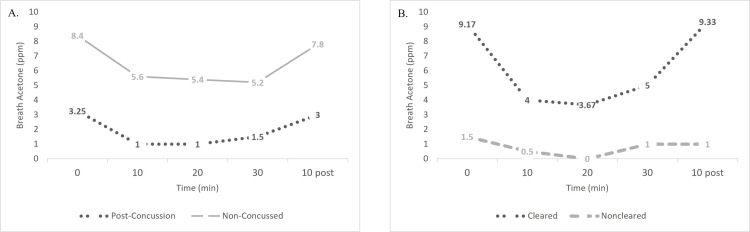
Average Breath Acetone Values Throughout Exercise Challenge Breath acetone was measured at rest (0 min), after 10 minutes of exercise, 20 minutes of exercise, 30 minutes of exercise, and 10 minutes of rest. A: Average breath acetone of non-concussed participants and post-concussion participants. B: Average breath acetone of post-concussion participants comparing those cleared to participate in activities and those not cleared. Abbreviations: parts per million (ppm), minute (min)

During the exercise challenge, breath acetone was measured at five time points. These points were at seated rest (following a 10-minute washout period), 10 minutes into cycling, 20 minutes into cycling, 30 minutes into cycling, and after 10 minutes of seated rest following cycling. Average breath acetone values for post-concussion and control groups demonstrate similar trends across the exercise challenge, with the post-concussion individuals expressing the visually lower acetone values throughout (Figure 6A). Within post-concussion participants, visual comparisons of cleared and non-cleared individuals demonstrated differences where cleared participants started at a higher resting level and exhibited a sharper curve over the progression of exercise before returning to their resting value (Figure 6B). However, when resting values were compared statistically between cleared and non-cleared individuals with the Mann-Whitney U test, they were not significant (P=0.20), nor was there a difference between the values after 30 minutes of exercise (P=0.40) or when subtracting the value at 30 minutes from the value at 10 minutes post-exercise (P=0.20). Mann-Whitney U tests did not support the hypothesis that breath acetone could distinguish prognostically between individuals who were later cleared on their own accord or not cleared (P=0.80).

Baseline and post-concussion participant

**Figure 7:**
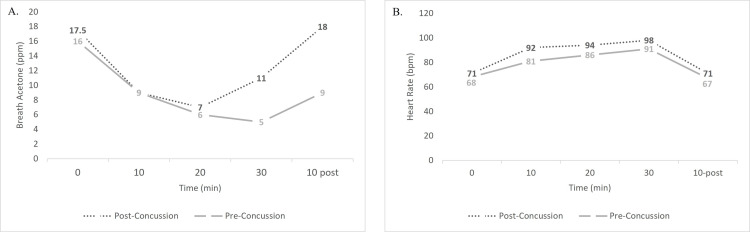
Comparison of Pre- and Post-concussion Measures in Same Participant During a 30-minute Exercise Challenge A: breath acetone values across an exercise challenge pre-concussion and post-concussion (N=1). B: heart rate values across an exercise challenge pre-concussion and post-concussion (N=1). Measures were done in the same participant. Abbreviations: parts per million (ppm), beats per minute (bpm), minute (min)

One participant completed the exercise challenge at the start of the season while non-concussed and again after sustaining a concussion. This participant completed the exit exercise challenge when cleared to participate in contact activity at 14 days post-concussion and is the only participant with both a non-concussed and post-concussion exercise test. Visually the participant demonstrates similar values at rest and throughout 20 minutes of cycling; however, after 20 minutes of exercise the breath acetone values begin to diverge (Figure 7A). These levels of breath acetone diverge, despite heart rate maintaining similarity throughout the pre- and post-injury tests (Figure 7B).

## Discussion

This investigation aimed to explore the diagnostic potential of breath acetone as a VOC in concussive injuries. Differences between concussed and non-concussed participants following injury and during recovery must be understood to fully grasp the diagnostic potential of volatile organic compounds. Results of this study showed that the highest recorded breath acetone value during the first week after concussion was significantly greater than that of the non-concussed individuals. The secondary hypothesis of this study was that there would be no differences in non-concussed and concussed individuals at the time of the exit exercise test, which took place when individuals were cleared to return to activity or 28 days after enrollment. Results of this study supported this hypothesis by showing that there were no differences in breath acetone between concussed and non-concussed athletes during the exit exercise test. Based on these initial findings, there is some support for the hypothesis that volatile organic compounds, and potentially breath acetone, could be an objective measure of concussion, but more research is needed in larger and more diverse athlete populations to identify the optimal timing of assessment and to establish a normative versus concussed range of concentrations.

The primary hypothesis of this investigation was that a significant statistical difference in breath acetone between non-concussed and concussed individuals exists during the injury presentation. The highest value obtained during the first seven days post-concussion was significantly greater than non-concussed individuals at rest and when compared to everyone’s own values at clearance or 26 days post-concussion. It has been established that the timing of the neurometabolic cascade differs depending on age and severity [15]. Though the acute acetone values (day two and day three) were not statistically different to the control participants, there is reason to believe the timing of the cascade could be unique to the individual and therefore single-day or average comparisons may not be capable of detecting values outside of the norm. In support of this, research has shown that when dietary changes such as calorie restriction or fasting are used to intentionally change energy substrate utilization, breath acetone slowly increases across a range of three to eight days before establishing a new steady state [22]. Individual’s preference as to the use of ketones over glucose prior to concussion may affect the onset of the switch in energy substrates when glucose is unable to be utilized. Results of the Kruskal-Wallis analysis and subsequent post-hoc multiple comparisons suggest a significant difference between the highest value measured in the first week and all time points considered (non-concussed controls, day three post-concussion, and resting at exit or clearance) except for day two post-concussion. This supports the need for immediate and consistent follow-up measurements to characterize the response curve. There is potential that measures earlier than two days post-concussion could be similarly statistically different as the highest value recorded in the first week was to controls’ resting. Additionally, further research with more participants is necessary to identify if differences in prognosis (i.e., time until return to activity) can be determined during the early stages of physiologic change demonstrated by breath acetone alterations.

The secondary hypothesis of this investigation was there would be no differences in breath acetone between non-concussed and post-concussion individuals throughout an exercise challenge conducted when concussed individuals were either cleared to participate in contact activity or approximately 28 days following obtainment of study consent. The results of this study demonstrated statistically that there were no differences in breath acetone values immediately prior to, during, or after the exercise challenge. In humans, the stage of decreased glucose utilization and dysfunctional oxidative metabolism typically lasts two to four weeks [14]. It was hypothesized there would be no differences in breath acetone due to the consequences of the neurometabolic cascade being resolved by the time the exercise test was completed, or because, as athletes were cleared, they were thought to have recovered physiologically. Comparisons between the highest value measured in the first week post-concussion with those measured at the exit test showed statistically significant differences, suggesting there is a difference between the first week of concussion recovery and clearance or four weeks post-concussion. Interestingly, visual differences apparent in graphed comparisons of the non-concussed and concussed participants throughout the exercise challenge showed that the control participants demonstrated higher breath acetone levels than the participants who had sustained a concussion. Both exhibited similar patterns across the exercise challenge, but the difference was maintained throughout. It is unclear why this occurred, and further research may be warranted to understand whether metabolic efficiency or glycogen stores are impacted following concussion.

Half (N=3) of the participants were cleared to return to activity prior to reaching 28 days post-concussion and completed the exercise test at time of clearance. The other half (N=3) completed the exercise test approximately 28 days following injury. The small sample size limits statistical analyses, especially as one participant (N=1) was unable to complete the same exercise challenge due to severity of symptoms, and thus only three cleared and two non-cleared participants were able to be compared. Statistical tests did not demonstrate differences, but visually, a divergence was present after exercise ceased and the 10-minute recovery period commenced. The original intention of this exploratory investigation was to establish comparisons of each concussed individual by their own baseline. However, due to COVID-19 limitations baseline assessments were only able to be collected in six individuals, and only one individual had a baseline and sustained a concussion. A paired t-test demonstrated significant differences between the highest first week value and that at clearance/26-day. At clearance and 26 days post-concussion individuals are thought to have resolved the physiologic consequences of concussion and thus could be termed as “non-concussed.” This individual comparison considers the individual variation and supports the notion that individuals have returned to a metabolic norm at rest. Ideally, future research would establish normative baseline measures on individuals so that pre-injury values may be used for analysis.

In the single participant with pre- and post-concussion exercise challenges, visual differences in breath acetone appeared 20 minutes into the exercise challenge and further diverged throughout exercise recovery despite heart rate remaining relatively similar. These results suggest that although a medically cleared individual may return to their metabolic normal at rest, physiologic consequences of the injury may reappear after exercise. The notion that individuals may be clinically cleared to return to play while still exhibiting physiologic dysfunctions is not a new one. Recent studies on physiologic consequences such as intracortical inhibition manifesting in motor deficits or musculoskeletal injury risk have demonstrated impairments despite an individual no longer experiencing signs and symptoms [23-26]. A larger sample of individuals before and after concussion with consideration of time to return to activity may provide more enlightenment in this area with breath acetone as a possible metric. Additionally, following participants for an extended period after return to activity clearance could provide answers to whether individuals have indeed returned to normative physiologic values when symptoms and presentation of the injury have resolved. Testing breath acetone throughout the return process could help personalize the introduction of activity after injury.

Though historically, concussed athletes were restricted from activities until symptoms resolved based on the international consensus recommendations, new research has shown light exercise is safe and improves symptom outcomes [1,27], and thus standardized rehabilitation plans have become personalized to include light activity. At this university, light activity included low-intensity and no-resistance cycling. These light activities were not tracked for the purposes of this study. In the future, measurements of VOCs should be taken throughout those short bouts of cycling while participants are symptomatic to see if differences between concussed and non-concussed are apparent during exercise when conducted earlier in the recovery process.

The results of this study were consistent with the recent findings in military sub-concussive exposures by Miller et al. (2022) [11]. Acetone seems to be impacted following concussion and should be included in any biomarker assessments utilizing volatile organic compounds. Previous research did not focus on the exhalation of breath acetone and ability to measure with a portable device, whereas this research demonstrates the ability of these breathalyzer devices to detect sensitive changes. Breathalyzer devices measure acetone and sometimes incorporate other volatile organic compounds in their measurements in a unique algorithm dependent on the device manufacturer. Due to the potential for other volatile organic compounds to falsely raise the values obtained by the breath acetone device, these results should be seen as demonstrating changes in volatile organic compounds as a whole and not solely breath acetone. Repeated measures within individuals utilizing the same device are suggested to limit the variability on the collection method.

The study was limited due to the small sample size of both the non-concussed and concussed individuals. Though efforts were made to recruit football and men’s/women’s soccer players prior to the start of the season, COVID-19 procedures made it difficult and burdensome to get access to a multitude of players. It was not possible to attain acute breath acetone measures within 24 hours of injury due to the limited availability of research staff, but future larger studies should endeavor to do this so that the trajectory of change in breath acetone following injury can be recorded. Additionally, concussive injuries were not always witnessed by medical staff and often were not reported by the athletes themselves. Of the current study participants, two had concussions reported by other teammates, and thus a delay in testing occurred. Future research should attempt to collect regular breath measurements to detect variants from a norm and immediate same-day measurements following a potentially concussive hit to better inform the diagnostic capabilities of volatile organic compounds. Recommendations cannot be made from these initial findings due to the small sample size of this exploratory study. However, the results from these comparisons demonstrate a need to investigate metabolic markers, further for their potential capabilities to diagnostically differentiate individuals.

## Conclusions

Establishing novel non-invasive biomarkers that may be evaluated sideline is important to the progression of concussion diagnostic research. The current investigation exploring volatile organic compounds and breath acetone as a potential diagnostic biomarker showed promise during the first week of concussion. Additionally, it revealed that though individuals may be symptomatically cleared, remaining physiologic consequences may be unearthed during a return to activity. The current study was limited due to the COVID-19 pandemic; however, based on these results, future research into energy substrate utilization as a marker of concussion should be conducted within larger sample sizes to account for variation and with immediate and continuous testing following a potentially concussive hit. This study used breath acetone devices that may unintentionally incorporate other volatile organic compounds. Further research should be conducted to differentiate compounds discovering which are providing the most influence.

## References

[1] Patricios JS, Schneider KJ, Dvorak J (2023). Consensus statement on concussion in sport: the 6th International Conference on Concussion in Sport-Amsterdam, October 2022. Br J Sports Med.

[2] Chandran A, Boltz AJ, Morris SN, Robison HJ, Nedimyer AK, Collins CL, Register-Mihalik JK (2022). Epidemiology of concussions in National Collegiate Athletic Association (NCAA) Sports: 2014/15-2018/19. Am J Sports Med.

[3] Kamins J, Giza CC (2016). Concussion-mild traumatic brain injury: recoverable injury with potential for serious sequelae. Neurosurg Clin N Am.

[4] Tator CH (2013). Concussions and their consequences: current diagnosis, management and prevention. CMAJ.

[5] Daly E, Pearce AJ, Finnegan E (2022). An assessment of current concussion identification and diagnosis methods in sports settings: a systematic review. BMC Sports Sci Med Rehabil.

[6] Rosenbloom C, Chatterjee R, Chu W, Broman D, Okholm Kryger K (2022). Sport-related concussion return-to-play practices of medical team staff in elite football in the United Kingdom. Sci Med Footb.

[7] Di Pietro V, O'Halloran P, Watson CN (2021). Unique diagnostic signatures of concussion in the saliva of male athletes: the Study of Concussion in Rugby Union through MicroRNAs (SCRUM). Br J Sports Med.

[8] Fedorchak G, Rangnekar A, Onks C (2021). Saliva RNA biomarkers predict concussion duration and detect symptom recovery: a comparison with balance and cognitive testing. J Neurol.

[9] Issitt T, Wiggins L, Veysey M, Sweeney ST, Brackenbury WJ, Redeker K (2022). Volatile compounds in human breath: critical review and meta-analysis. J Breath Res.

[10] Caravati EM, Anderson KT (2010). Breath alcohol analyzer mistakes methanol poisoning for alcohol intoxication. Ann Emerg Med.

[11] Miller MR, DiBattista A, Patel MA (2022). A distinct metabolite signature in military personnel exposed to repetitive low-level blasts. Front Neurol.

[12] Giza CC, Hovda DA (2014). The new neurometabolic cascade of concussion. Neurosurgery.

[13] Greco T, Vespa PM, Prins ML (2020). Alternative substrate metabolism depends on cerebral metabolic state following traumatic brain injury. Exp Neurol.

[14] Giza CC, Hovda DA (2001). The neurometabolic cascade of concussion. J Athl Train.

[15] Prins ML, Hovda DA (2009). The effects of age and ketogenic diet on local cerebral metabolic rates of glucose after controlled cortical impact injury in rats. J Neurotrauma.

[16] Alkedeh O, Priefer R (2021). The ketogenic diet: breath acetone sensing technology. Biosensors (Basel).

[17] Kalapos MP (2003). On the mammalian acetone metabolism: from chemistry to clinical implications. Biochim Biophys Acta.

[18] Xue Y, Thalmayer AS, Zeising S, Fischer G, Lübke M (2022). Commercial and scientific solutions for blood glucose monitoring-a review. Sensors (Basel).

[19] Saslow LR, Moskowitz JT, Mason AE (2020). Intervention enhancement strategies among adults with type 2 diabetes in a very low-carbohydrate web-based program: evaluating the impact with a randomized trial. JMIR Diabetes.

[20] Akturk HK, Snell-Bergeon J, Pyle L, Fivekiller E, Garg S, Cobry E (2021). Accuracy of a breath ketone analyzer to detect ketosis in adults and children with type 1 diabetes. J Diabetes Complications.

[21] Fuke N, Ushida Y, Sato I, Suganuma H (2023). Inter-day variation in the fasting plasma lipopolysaccharide concentration in the morning is associated with inter-day variation in appetite in Japanese males: a short-term cohort study. Metabolites.

[22] Anderson JC (2015). Measuring breath acetone for monitoring fat loss: review. Obesity (Silver Spring).

[23] Nordström A, Nordström P, Ekstrand J (2014). Sports-related concussion increases the risk of subsequent injury by about 50% in elite male football players. Br J Sports Med.

[24] Miller NR, Yasen AL, Maynard LF, Chou LS, Howell DR, Christie AD (2014). Acute and longitudinal changes in motor cortex function following mild traumatic brain injury. Brain Inj.

[25] Lynall RC, Mauntel TC, Padua DA, Mihalik JP (2015). Acute lower extremity injury rates increase after concussion in college athletes. Med Sci Sports Exerc.

[26] Ntikas M, Hunter AM, Gallagher IJ, Di Virgilio TG (2021). Longer neurophysiological vs. clinical recovery following sport concussion. Front Sports Act Living.

[27] Cordingley DM, Cornish SM (2023). Efficacy of aerobic exercise following concussion: a narrative review. Appl Physiol Nutr Metab.

